# Plasma zinc concentration and children's infectious diseases

**DOI:** 10.1186/1471-2334-13-S1-P62

**Published:** 2013-12-16

**Authors:** Nicoleta Negruț, Lucian Negruț

**Affiliations:** 1 Third Department of Pediatrics, Iuliu Hațieganu University of Medicine and Pharmacy, Cluj-Napoca, Romania; 2 Department of Neuroscience and Recovery, Faculty of Medicine and Pharmacy, University of Oradea, Romania; 3 West University of Timişoara, Romania

## Background

Zinc deficiency perturbs the immune response and generates higher susceptibility of the human body to infections.

## Methods

The study’s primary outcome was the plasma concentration of zinc. The colorimetric method with Br-PAPS final point (CV% 0.98% - 4.64%) was used for the determination of the zinc level. Secondary outcomes were the prevalence of the acute infectious diseases in children and their relationship with plasma concentration of zinc. Data were processed using EPIINFO version 6.0.

## Results

During 2009-2011, 98 healthy children, 0-3 years old, from Bihor county, Romania, were enrolled in the study. A total of 96 children recruited were available for analysis. In the study group (n=96) the mean plasma concentration of zinc was 15.33±1.49 μmol/L.

Subjects with more than 3 episodes of acute respiratory infections/year had a statistically significant lower value of plasma concentration of zinc as compared to those who had less than 3 episodes/year (14.23±0.76 μmol/L versus 15.89±1.46 μmol/L, p<0.001, Student's t test).

There are no significant differences between those who had an episode of acute diarrhea/year compared with more than 1 episode/year (15.49±1.44 μmol/L versus 15.09±1.56 μmol/L, p=0.480, Student's t test).

Children with a history of parasitic infections had a mean plasma concentration of zinc similar to those without parasitic infections (15.01±1.32 μmol/L versus 15.42±1.14 μmol/L, p=0.450, Student's t test).

## Conclusion

In our study, the children aged 0-3 years showed no zinc deficiency. Smaller plasma concentrations of zinc were associated with more than 3 episodes of acute respiratory infections/year.

